# Narrative Review Explaining the Role of *HLA-A*, *-B*, and *-C* Molecules in COVID-19 Disease in and around Africa

**DOI:** 10.3390/idr16020029

**Published:** 2024-04-18

**Authors:** Lisa Naidoo, Thilona Arumugam, Veron Ramsuran

**Affiliations:** 1 School of Laboratory Medicine and Medical Sciences, College of Health Sciences, University of KwaZulu-Natal, Durban 4041, South Africa; lisa08naidoo@gmail.com (L.N.); cyboglona@gmail.com (T.A.); 2 Centre for the AIDS Programme of Research in South Africa (CAPRISA), University of KwaZulu-Natal, Durban 4041, South Africa

**Keywords:** host genetics, COVID-19, HLA

## Abstract

The coronavirus disease 2019 (COVID-19) has left a devasting effect on various regions globally. Africa has exceptionally high rates of other infectious diseases, such as tuberculosis (TB), human immunodeficiency virus (HIV), and malaria, and was not impacted by COVID-19 to the extent of other continents Globally, COVID-19 has caused approximately 7 million deaths and 700 million infections thus far. COVID-19 disease severity and susceptibility vary among individuals and populations, which could be attributed to various factors, including the viral strain, host genetics, environment, lifespan, and co-existing conditions. Host genetics play a substantial part in COVID-19 disease severity among individuals. Human leukocyte antigen (HLA) was previously been shown to be very important across host immune responses against viruses. HLA has been a widely studied gene region for various disease associations that have been identified. HLA proteins present peptides to the cytotoxic lymphocytes, which causes an immune response to kill infected cells. The HLA molecule serves as the central region for infectious disease association; therefore, we expect HLA disease association with COVID-19. Therefore, in this narrative review, we look at the HLA gene region, particularly, HLA class I, to understand its role in COVID-19 disease.

## 1. Introduction

Severe acute syndrome coronavirus 2 (SARS-CoV-2) causes COVID-19, which led to the most dreadful pandemic [1]. Coronavirus is a respiratory virus that can cause symptoms like the common cold and potentially lethal inflammation in extra-pulmonary organs [2]. COVID-19 outcomes, severity, and symptoms vary due to age, comorbidities, living conditions, viral genetics, genomics, blood groups [3,4], and host genetics involved in antiviral defense mechanisms and pathogenesis [5,6]. COVID-19 severity correlates with lymphopenia, cytokine storm [7], and exaggerated immune response [8]. 

COVID-19 began in Wuhan, China [9,10], and quickly reached Europe and the US [11,12], followed by various other countries around the world. However, in Africa, with a population of more than 1.2 billion people, COVID-19 infections and deaths have been relatively low, especially in malaria-endemic regions [13]. The overall death rate in Africa reduced from 2019 to 2020 and 2020 to 2021. The lower COVID-19 incidences have been attributed to the lack of COVID-19 testing, early government interventions, population distribution, social distancing, habitation ecology, demographics, existing medical conditions, innate immune memory, genetics, and larger sociocultural dynamics [14]. This also includes Africa having a younger population, public support, a favorable climate, good healthcare systems, and quick action [15]. Despite Africa being a developing and poorer continent, it was suggested that Africa had implemented additional public health guidelines compared to other nations and that previous involvement with human HIV, Ebola, TB, etc., left them more equipped to deal with COVID-19. Africa might have also had more time to prepare and respond to COVID-19 with well-developed guidelines compared to other countries. Sub-Saharan Africa has one of the highest rates of endemic infectious diseases, suggesting an uncommon response to COVID-19 [14,16,17]. This requires the need to establish the basis of Africa’s differential response during the pandemic. Host genetics could be the reason behind most of the SARS-CoV-2 susceptibility and COVID-19 severity variation between patients.

Human leukocyte antigens (HLAs) are major genes of the major histocompatibility complex (MHC), they play an integral part in presenting antigens to T cells, allowing the identification of foreign proteins from pathogens involved in various infectious diseases. HLA genes have been studied in various viral infections, they are beneficial to the immune response against viruses and related to vaccines [18]. It is crucial to understand the impact the HLA region has on individuals’ or populations’ responses to SARS-CoV-2. Genome-wide association studies, known as GWAS, showed genetic variants involved in immunological processes associated with SARS-CoV-2 susceptibility and COVID-19 severity [12,19]. Therefore, HLA genetic variations might contribute significantly to the variation in the immunological reaction to COVID-19 and might be associated with cytokine storm and, in turn, the variation in SARS-CoV-2 susceptibility and COVID-19 severity.

The 02HLA genes are located on the short arm of chromosome 6 (p21.3) [20]. The HLA molecule is found on the cell surface of most cells. HLA molecules are important in the adaptive immune response. It mediates specific attacks on infected cells and antibody production. HLA classical class I molecules are made up of *HLA-A*, *HLA-B*, and *HLA-C*, and they comprise two noncovalently bound polypeptide chains. Nucleated cells express HLA class I molecules [21]. The HLA gene codes for the polymorphic alpha chain, while chromosome 15 includes the nonpolymorphic beta-2 microglobulin chain gene. HLA class I molecules have endogenic peptides, comprising those that are virus-originated [22]. Class I antigens present foreign peptides that are identified by CD8 T cells [20,23]. HLA class II is categorized into three groups, *HLA-DR*, *HLA-DQ*, and *HLA-DP*. They are heterodimers made up of α and β chains, and display peptides produced in endosomes from presenting cells to CD4 T cells [20]. Antigen-presenting cells, such as macrophages, dendritic cells, and mature B lymphocytes, express HLA class II molecules. Intestine and lung epithelial cell surfaces also express HLA class II molecules [24]. HLA is the most polymorphic human gene [25]. There are more than 30,000 HLA alleles that have been identified, which code for approximately 18,000 different proteins [26]. However, there have been various database updates according to Rigen et al., 2023, such as removals and additions of new papers [27]. Most of the HLA mutations occur in the exons. These mutations encode for the peptide-binding groove and T-cell receptor interactions [28]. Genetic variations affect HLA geometry, hydrophobicity, charge distribution, and peptide interactions. Different HLA molecules may have unique peptide binding abilities to B-cell and T-cell receptors, known as immunoglobulins. Varying genotypes of HLAs in individuals might deviate in peptide presentation and immune responses [29]. HLA genetic polymorphisms affect the disease severity of RNA and DNA viruses, for example, influenza H1N1 [30], Hantaan [31], SARS-CoV-1 [32], HCV, HBV, HIV, hepatocellular carcinoma, liver cirrhosis [21,33], and bacterial infections such as tuberculosis [34]. Therefore, HLA studies could be valuable in determining which genes contribute to severe COVID-19 in patients. The HLA class I genes are required in developing a specific immunological response to viral infections. HLA-A*68 was shown to be associated with protection against COVID-19 severity and fatal outcomes [35]. In this narrative literature review, we will look at HLA class I genes that are COVID-19-associated. We will also discuss polymorphisms in this region that impact the COVID-19 disease outcome.

## 2. Selection Criteria

**Figure 1:**
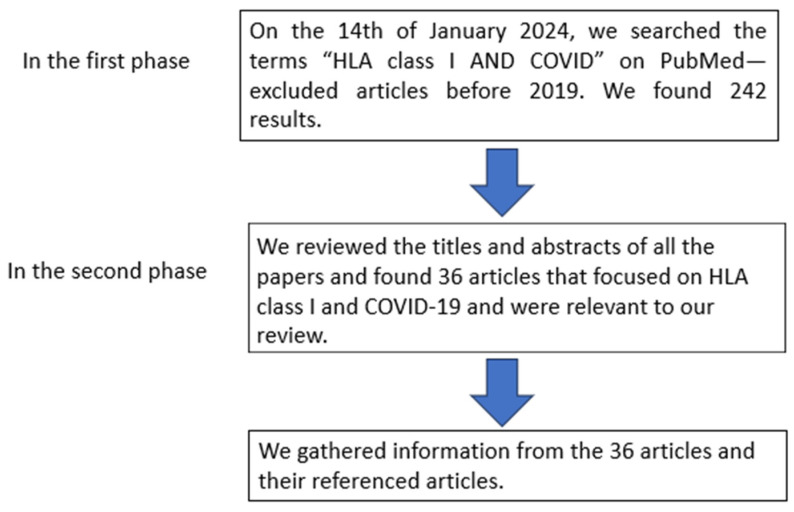
Flow diagram of the selection criteria of the article.

Articles were selected using the following criteria for our literature review. In the first phase, on 14 January 2024, we searched the terms “HLA class I AND COVID” on PubMed and excluded all articles before 2019. We found 283 results. In the second phase of the selection, we analyzed the titles and abstracts of all the articles and found 18 articles that focused on HLA class I and COVID-19. We retrieved information from the 36 articles and their referenced articles. This is summarized in Figure 1 below. Information regarding the gene, gene effect, ethnicity, no. of samples, and the *p*-value used in each study reviewed in this article are summarized in Table 1.

## 3. The Role of HLA in COVID-19

HLA antigens could be a valuable contributing factor in SARS-CoV-2 outcomes [79,80]. Studies have shown specific HLA alleles correlated with the risk of SARS-CoV-2 infectivity, COVID-19 disease progression, and vaccine responses. Individuals with different HLA profiles but the same antigen may result in unique T-cell-mediated immune responses because they have contrasting numbers of specific HLA antigen-derived epitopes. Some studies distinguish the viral antigens presented by specific HLAs [79,80,81]. Understanding how T-cell reactivity and antigen presentation are associated with HLA, and the immune mechanisms responsible for different host immune reactions to SARS-CoV-2, may assist researchers to develop strategies to alleviate COVID-19 [82]. The HLA variations determine the antigen presentation [82]. Studies have shown the importance of peptide magnitude, specificity, and the quality of cellular and humoral immune responses. In silico research, analysis has been conducted on the binding ability among peptides of SARS-CoV-2 and diverse genotypes of HLA class I [42,47,61,83]. HLA studies have shown insight into viral susceptibility in different populations. Hence, studies have uncovered HLA alleles that contributed to susceptibility or resistance to COVID-19 in different ethnic groups. While other studies did not show a relationship between HLA polymorphism or haplotypes and COVID-19 susceptibility and resistance [68]. Therefore, it is important to further analyze these HLA alleles that could be targeted in therapeutic strategies to alleviate the COVID-19 disease and be aware that this might vary between ethnicities.

### 3.1. HLA-A

Hernandez-Dono et al., 2022, studied the relationship between HLA alleles and severe COVID-19 in Tapachula, Mexico. This study consisted of 146 Mexicans. The patients were categorized according to their outcome (deceased or recovered) and severity (moderate or severe), and exposed uninfected participants were included. This study showed that the HLA-A*68 allele protected against severe COVID-19 and fatal outcomes. COVID-19 severity and fatal outcomes in Tapachula, Chiapas, were predominantly dependent on the absence of resistance than HLA susceptibility alleles. There was a significant statistical difference in HLA-A*68 among COVID-19-infected individuals and exposed uninfected individuals, and severely infected COVID-19 individuals and exposed uninfected individuals. HLA-A*68 was absent among severe COVID-19 and COVID-19 patients. In carriers, this allele conferred 2.4 times more severe SARS-CoV-2 infection resistance. It also protected against deadly SARS-CoV-2 outcomes 3.3 times more in Tapachula, Chiapas, and mestizo participants [35]. In the same study, HLA-A*01 was associated with the risk of COVID-19 fatal outcomes. There was a significant statistical difference between deceased patients and recovered patients [35]. This study showed two different HLA-A genes that had different associations with COVID-19. A western Indian population showed HLA-A*01 was more prevalent in controls than COVID-19-infected individuals (*p* = 0.011), while HLA-A*02 was prevalent with COVID-19-infected individuals of varying severity (*p* < 0.001) [37]. Similarly, HLA-A*01 was associated with low ferritin, which was associated with low severity (*p* = 0.016) [69]. In Russia, HLA-A*01:01 was expressed at lower in severe pneumonia and indicated a protective factor for severe COVID-19 [38]. HLA-A*02:01 was associated with the risk of symptomatic COVID-19 (*p* = 0.03), and HLA-A*02:05 was linked to severe respiratory infection risk (*p* = 0.04) [40]. These genes need to be further studied in other populations to determine if their effect varies.

A bioinformatics analysis screened for possible SARS-CoV-2 epitope sequences for HLA. This study identified two epitopes that are nonstructural proteins in the open reading frame (ORF) that displayed a compelling binding affinity for HLA-A*24:02, HLA-A*02:06, and HLA-A*02:01 in a Japanese cohort. These epitopes had the highest population coverage at 83%. Therefore, they were regularly accessible and applicable to a bigger population [41]. Regrettably, this study’s mathematical estimates need to be better defined with regard to immunogenetic traits and clinical and experimental estimates [46]. 

HLA-A*11:01 was associated with severe disease in a Japanese cohort, OR = 3.41 and *p* = 0.003 [46]. A study with 190 individuals from Japan with moderate to severe COVID-19 observed an association between HLA-A*11:01 and COVID-19 severity (OR = 2.26) (*p* = 0.013) [46]. *HLA*-A*11:01 significantly correlated with COVID-19 severity, hospital admission, and fatality after adjusting for sequential organ failure assessment (SOFA) or acute physiology and chronic health evaluation II (APACHE-II) when compared with mildly infected COVID-19 patients [36,45,46,47,71]. SOFA is a score used to anticipate mortality in septic patients, while APACHE II is a score of the disease severity categorization taken during the initial 24 hours after hospital admission. Similarly, another study using logistic regression analysis showed that HLA-A*11 was linked with increased mortality, after regulating for APACHE-II (*p* = 0.02) or SOFA (*p* = 0.04). They found an increased frequency of HLA-A*11 (*p* = 0.051) in the deceased than in survivors [34]. Toyoshima et al. showed that HLA-A*11:01 was defensive against SARS-CoV-2 susceptibility and COVID-19 fatality when compared to the global database for allele rate, infection, or death [71], which were inconsistent with research that compared allele incidence and effect at an individual level [45,46,47]. Analysis of inferences from bioinformatics and practical applications should be undertaken with caution because different methods may result in differing and incomparable results. HLA-A*11 was associated with weak evolution in other infectious diseases [34,84]. The HLA allele data from next-generation sequencing (NGS) of 332 patients from China who were admitted to a hospital showed the difference between allele frequency among individuals severely and moderately infected with COVID-19 (*p* = 0.009) [45]. HLA-A*11:01 allele could protect against infection [71]. Another study with 73,000 Israeli individuals, including 6413 SARS-CoV-2-infected individuals and 181 COVID-19-hospitalized individuals, showed no significant association with common HLA alleles [85]. HLA-A*11:01 had a significantly negative association with SARS-CoV-2 susceptibility and mortality in 21 countries. However, this was insignificant for the death rate when modified for the S 614G variant. Studies of HLA-A*11:01 were conflicting regarding SARS-CoV-2 susceptibility. HLA-A*11 possibly confers SARS-CoV-2 susceptibility in different populations, such as Chinese, Indian, and Asian individuals born in the United Kingdom; Hispanic; and Black [39,47]. In the Spanish population of 5943 controls and 9373 COVID-19-infected individuals, HLA-A*11:01 was associated with COVID-19 severity (*p* = 0.033) [48]. These studies suggest that HLA-A*11:01 could have varying effects on COVID-19 in different ethnicities. HLA-A*11:01 might be a possible therapeutic target in some populations. 

HLA-A*02:01 positively correlated with increased risk of COVID susceptibility and mortality [42], due to its low SARS-CoV-2 antigen presentation ability. However, HLA-A*02 showed protection against susceptibility and death [39]. Shkurnikov et al. (2021) showed that HLA-A*02:01 and *HLA*-A*03:01 were associated with low COVID-19 risk [44]. HLA-A*02:01 showed contrasting results between these studies. Therefore, more research is required on this allele to understand its role in COVID-19 disease. Haplotype HLA-A*02:01g~B*18:01g~C*07:01g~DRB1*11:04g negatively correlated with COVID-19 and, therefore, might also be protective against infection [56]. HLA-A*24:02:01 was associated with SARS-CoV-2 susceptibility and severity among Chinese [45,51]. The HLA-A*1 allele was found in four out of five South Han Chinese COVID-19 patients. This allele has been associated with diabetes, a risk factor for COVID-19 [51]. HLA-A*24:02 and HLA-A*26:01 [44] may worsen the COVID-19 outcome. Contrastingly, in Ecuador and Madrid, HLA-A*24:02 was linked to protection from severe COVID-19 [50]. A study with a Brazilian population showed that HLA-A*23:01, HLA-A*24:02, HLA-A*26:01, HLA-A*30:02, HLA-A*31:01, and HLA-A*68:01 were associated with protection against COVID-19 [49,77]. *HLA*-A*30:02 alleles among African Americans [52] were more significantly susceptible to SARS-CoV-2 infection. HLA-A*30:02 is prevalent in Africa and Sardinia [66,86,87]. Larger sample sizes are required to validate the role of these *HLA-A* alleles on COVID-19. 

An *HLA-A*-genotyping study was performed with 72 COVID-19-infected individuals and 3886 well individuals in the control group. The frequency of HLA-A*32 (*p* = 0.004) was higher in the control group when compared to the COVID-19-infected individuals, and HLA-A*03 (*p* = 0.047), HLA-B*39 (*p* = 0.02), and HLA-C*16 (*p* = 0.02) alleles were more prevalent in COVID-19-infected participants compared to healthy individuals; but, after multiple assessments modification, the *p*-values were insignificant. This might be attributed to the small COVID-19-positive cohort. In another study, HLA-A*03 was associated with risk, and HLA-A*32 was associated with protection. After correction, only HLA-A*03 remained significant [43]. A study with a Brazilian population showed that HLA-A*01:01, HLA-A*02:01, and HLA-A*03:01 were associated with protection against COVID-19 [49,77].

HLA class I molecules have varied reactivity to cytotoxic T lymphocytes. Another study showed that weak *HLA-A* and B haplotypes are associated with deaths and COVID-19 severity [88]. Additional studies are required to authenticate the impact of these *HLA-A* alleles between healthy and infected individuals. 

### 3.2. HLA-B

HLA-B*08:01 and HLA-B*08 correlated with raised COVID-19 risk and mortality [54,56,57,71]. Pisanti et al. suggested that HLA-B*18:01 and haplotype HLA-A*02.01g-B*18.01g-C*07.01g-DRB1*11.04g protect against COVID-19 occurrence and mortality [56]. HLA-B*18:01, HLA-B*35:03, *HLA-38:01*, HLA-B*44:02, and HLA-B*51:01 were associated with protection against COVID-19 among the Brazilian population [49,77]; whereas HLA-B*44, *HLA*-B*44:02, and HLA-B*58:01 were associated with greater risk in population reports for SARS-CoV-2 susceptibility, COVID-19 severity, and death. However, this was not reflected in laboratory studies [39,54,57,58,66,72]. In another study, HLA-B*44:02 was associated with a risk of respiratory hospitalization (*p* = 0.008) [40]. Varying study designs should be compared with caution, and other contributing factors should be taken into account. 

Nguyen et al. (2020) showed that HLA-B*15:03 was protective against COVID-19 by efficiently presenting conserved peptides of SARS-CoV-2 to T cells, and found it had one of the highest binding affinities to SARS-CoV-2 peptides [61]. In this study, mathematical predictions required experimental and clinical evaluation and participants with immunogenetic characteristics. *HLA-B* frequency data were acquired from the Allele Frequency Net Database [89] for 805 different populations from 101 countries. There was a robust linkage disequilibrium between HLA-B*15:01 and HLA-DRB1*04:01; this correlated considerably with infected European individuals who presented no symptoms [62]. In a western Indian population, HLA-B*15 was associated with protection against COVID-19 (*p* = 0.008), while HLA-B*40 was associated with mild COVID-19 infections (Pc = 0.03) [37]. Similarly, in an Egyptian population, HLA-B*15 was significantly associated with protection (*p* < 0.001) [60]. In a study consisting of 82 COVID-19-infected individuals from China, the frequencies of HLA-B*15:27 and HLA-B*40:06 were statistically higher in COVID-19-infected individuals than in healthy controls [59]. However, this study did not have enough power to find a substantial association between HLA polymorphism and COVID-19 susceptibility. Studies with higher power and a bigger cohort are required to decipher the role of *HLA-B* alleles in COVID-19 disease. Cheranev et al. (2023) showed that statistically significant alleles joined into haplotypes HLA-B*27:02:01G and HLA-C*02:02:02G, and HLA-B*14:02:01G and HLA-C*08:02:01G were prevalent in deceased patients and survivors, due to linkage disequilibrium, respectively [90]. In Spain, HLA-B*14:02 was associated with a reduced risk of COVID-19 (*p* = 0.006) [48].

In Egypt, a study with 69 COVID-19 patients showed that HLA-B*41 and HLA-B*42 were associated with severe COVID-19 [60]. HLA-B* 46:01 was associated with SARS-CoV-1 severity in an Asian population and SARS risk [32]. HLA-B*46:01 has a low binding affinity to SARS-CoV-2 peptides, indicating that individuals with HLA-B*46:01 might have increased COVID-19 susceptibility [61]. HLA-B*46:01 [61] and HLA-B*07 displayed a role in susceptibility among a cohort consisting of multiple ethnicities [54]. These estimates were not evaluated clinically and by experimentation and had insufficient immunogenetic traits. Similarly, HLA-B*46:01 was associated with SARS-CoV-2 severity [32,61,91]. HLA-B*46:01 is uncommon in the United States (US), Switzerland, and Spain [91]. HLA-B*46:01 was absent in the data comprising Europeans. Analyses established that most of the allele frequencies in the German cohort were comparable in cohorts from Switzerland, the US, and Spain. The SARS-CoV-1 outbreak revealed that HLA-B*46:01 [32] and HLA-B*07:03 were associated with disease [55]. HLA-B*46:01 is significantly associated with SARS-CoV-2 susceptibility among Singaporeans, Chinese, Vietnamese, and Taiwanese, except for children of mixed ethnicities [32]. Wang et al. and Gutierrez-Bautista et al. (2022) showed that HLA-B*46:01 is prevalent in mild COVID-19 compared to severe COVID-19, and it does not present SARS-CoV-2 peptides well [68]. SARS-CoV-2 is approximately 77% similar to the SARS-CoV-1 genome [92], so it is typically acceptable to assume similarities in the host immune reaction to the SARS-CoV viruses. 

The HLA-B*22 serotype is a possible marker for SARS-CoV-2 risk [64]. Barquera et al. (2020) showed five HLA-B*22 alleles (HLA-B*54:01, HLA-B*55:01, HLA-B*55:07, HLA-B*55:12, and HLA-B*56:01) possessed the lowest binding ability to SARS-CoV-2, indicating that HLA-B*22 is associated with SARS-CoV-2 susceptibility [70]. In another study, HLA-B*55 and HLA-B*58 were associated with protection; however, after multiple corrections, this was insignificant [43]. HLA-B*54:01, HLA-B*56:01, and HLA-B*56:04 were associated with COVID-19 patients when compared to Hong Kong Chinese Cord Blood Registry controls (*p* > 0.05) [93]. The HLA-B*27 serotype might regulate SARS-CoV-2 infection [64] and is linked with infectivity and protection against all strains of SARS-CoV-2 [94]. The change in immune homeostasis could be involved in coronavirus pathogenesis. Yung et al. (2020) performed a study with 190 Chinese participants with COVID-19. They showed a correlation between the HLA-B*22 serotype and SARS-CoV-2 infection (*p* = 0.032, OR = 1.71) [64]. Epitopes of SARS-CoV-2 and HLA-A*02:06, HLA-B*52:01, and HLA-C*12:02 shared high binding affinity. Binding affinity studies highlight and put into perspective the impact of these HLA alleles on COVID-19. 

COVID-19 patients in a Chinese cohort showed HLA-B*51:01 alleles were significantly associated with severe COVID-19 [45]. This allele had quite reduced SARS-CoV-2 antigen presentation ability compared to other HLA class I molecules [42]. HLA-B*54:01 alleles might be responsible for protecting against COVID-19 infection [71]. HLA-B*15:01 was significantly associated with asymptomatic SARS-CoV-2 [63]. In Italy, HLA class I alleles could play a role in the differences in the extent of SARS-CoV-2 infection between North and South Italy. HLA allele frequency from a bone marrow donor registrar in Italy and the prevalence of SARS-CoV-2 infection in various districts were assessed. HLA-B*08, HLA-B*15:01, HLA-B*44, and HLA-B*51 positively correlated with COVID-19. However, HLA-B*14, HLA-B*18, and HLA-B*49 were inversely associated with COVID-19. HLA-B*44 alleles were found at a higher incidence in Italians from the north and were positively associated with COVID-19, after multiple regression models [58]. This epidemiological analysis shed light on specific HLA class I alleles that are not capable of presenting adequate virus-derived epitope peptides to initiate an acceptable SARS-CoV-2 immune response to offset infection. HLA-B*44 alleles are tolerant to SARS-CoV-2 infection in Italians [95,96]. HLA-B*44 inheritance is the cause of recurrent sinopulmonary infection susceptibility [97]. The analysis of HLA allele data from NGS of 332 hospitalized Chinese patients detected variations among mild and severe COVID-19 infections in individuals with HLA-B*51:01 (*p* = 0.007) [45]. Similarly, HLA-B*51 was associated with fatal COVID-19 in a South Asian population [69]. HLA-B*37:01 was associated with deceased COVID-19 individuals (*p* = 0.0331); therefore, it might be involved in severe COVID-19 disease outcomes [44,98]. Poulton et al. (2020) showed that HLA-B*44 might have a protective effect against SARS-CoV-2 infection when compared to controls. HLA-A*02, HLA-B*44, and HLA-C*05 are usually inherited together. This might cause a protective effect or effective immune response against COVID-19 [39].

Seven HLA haplotypes or alleles were identified as defensive against SARS-CoV-2 infection. In addition, five haplotypes or alleles correlated with enhanced susceptibility to SARS-CoV-2. HLA-A*30:02, HLA-B*14:02, and HLA-C*08:02 three-loci haplotypes were statistically significant after *p*-values were corrected. There was a strong correlation observed between this haplotype and COVID-19 disease severity [66]. Geographical epidemiology analysis showed significant variances in the incidence of two of the prevalent HLA haplotypes in Italians between North, Central, and South Italy, with HLA-A*01:01g (change in expression)-B*08:01g-C*07:01g-DRB1*03:01g (prevalent haplotype countrywide) showing a declining incidence gradient, and HLA-A*02:01g-B*18:01g-C*07:01g-DRB1*11:04g (second prevalent haplotype) showing a cumulative incidence gradient from Northern to Southern Italy. The haplotype division correlates with COVID-19 in Italians. HLA-A*01:01g-B*08:01g-C*07:01g-DRB1*03:01 is indicative of COVID-19 susceptibility, while HLA-A*02:01g-B*18:01g-C*07:01g-DRB1*11:04g might be associated with COVID-19 protection [56]. 

HLA-B*35 was significantly associated with severe COVID-19 in a study with 92 patients of 15 nationalities from the United Arab Emirates (*p* = 0.0051) [65]. Similarly, HLA-B*35 was more significantly associated with mild than severe COVID-19 in a South Asian population [69]. Farahani et al. (2021) and Shekarkar et al. (2020) observed a significant association between HLA-B*38 and disease susceptibility in the Iranian population [53]. In Spain, a study, conducted with patients from six ICUs observed higher COVID-19 infection rates among individuals with HLA-B*39, but these *p*-values were insignificant after multiple comparisons correction [62]. A total of 3886 healthy individuals and 72 COVID-19-infected individuals were genotyped for *HLA-B*. HLA-B*39 (*p* = 0.02) alleles were found more in COVID-19-infected individuals than in healthy individuals; yet, the *p*-values were insignificant after being adjusted for multiple comparisons. These studies included a small COVID-19 population, which might be the reason for the absence of significant differences. In an Ecuadorian population made up of 52 COVID-19-infected individuals and 87 controls, HLA-B*39 was associated with a risk of COVID-19 development [50]. 

### 3.3. HLA-C

High binding affinity was observed between epitopes of SARS-CoV-2 and HLA-C*12:02, which suggests a high immune response against COVID-19 [41]. A study with 82 COVID-19-infected Han individuals from Zhejiang found a statistically significant difference between HLA-C*07:29 in COVID-19 patients compared to controls; this allele was prevalent in COVID-19 patients [59]. All these individuals had moderate or severe COVID-19, had no critical conditions, donated plasma after recovery and were aged between 20 to 54. However, only one COVID-19-infected individual possessed a HLA-C*07:29, and none of the controls [59]. These findings should be proven in studies with greater sample sizes. A study with Chinese individuals showed that HLA-C*14:02 significantly correlated with severe COVID-19 [45]. HLA-C*03 was associated with high ferritin, which was associated with increased COVID-19 severity in the Saudi population [69]. Another study showed that HLA-C*01 and HLA-C*03 were positively related to the prevalence of SARS-CoV-2 infection. After multiple regression models, only HLA-C*01 alleles, which are common in northern Italy, were positively associated with COVID-19. This was established by a diverse provincial sub-analysis in Italy [58]. In this study, they genotyped *HLA-C* in 72 COVID-19-infected individuals and 3886 healthy individuals. HLA-C*01 alleles might be permissive to SARS-CoV-2 infection among Italians [58]. HLA-C*01 was more prevalent (*p* = 0.09) in the deceased than in survivors. In addition, the allele HLA-C*01 was associated with a higher death rate after modulating for SOFA (*p* = 0.04) or APACHE-II (*p* = 0.02) [36]. HLA-C*01 was formerly associated with the risk of other infectious diseases [99]. Tripathy et al. (2023) showed that HLA-C*01 was associated with mild COVID-19 infection (*p* = 0.004) [37]. There were more HLA-C*16 alleles (*p* = 0.02) found in COVID-19 patients compared to controls; nonetheless, all the *p*-values were insignificant after multiple comparisons adjustment [36]. HLA-C*05:01 was significantly associated with the risk of COVID-19 death [54]. HLA-C*05:01, HLA-C*07:01, HLA-C*08:02, HLA-C*15:02, and HLA-C*17:01 were associated with COVID-19 protection [49,77]. Among 69 COVID-19 patients from Egypt, HLA-C*16 and HLA-C*17 were associated with COVID-19 severity, while HLA-C*7 and HLA-C*12 were associated with protection from death [60]. HLA-C*08:02, HLA-C*12:03, and HLA-C*16:01 were more prevalent in mild COVID-19 than severe COVID-19 (*p* = 0.0014) in a Spanish Mediterranean Caucasian population [78]. In another Spanish population, HLA-C*08:02 was associated with a reduced risk of COVID-19 (*p* = 0.024) [48]. In Spain, a study consisting of ICU patients observed increased COVID-19 infection rates in individuals with HLA-C*16, but the *p*-values were insignificant after multiple comparisons correction. The small COVID-19 population in these studies could justify the insignificance [36]. 

Shkurnikov et al. (2021) observed that HLA-C*06:02 significantly correlated with COVID-19 mortality; therefore, it may be related to more severe COVID-19 outcomes [39]. On the contrary, HLA-C*05 was found more frequently among controls and, therefore, protects against SARS-CoV-2 infection. Although, this was not significant after multiple test corrections [39]. Additional studies are imperative to understand the role of HLA-C*05 in COVID-19.

HLA allele data from the NGS of 332 individuals from the Shenzhen Third Peoples’ Hospital, China revealed that HLA-C*14:02 was significantly prominent in severe compared to mild COVID-19 cases (*p* = 0.003) [45]. Further research will determine if this allele could be a potential target for therapeutics in a Chinese population. 

In Europeans, HLA-C*04:01 has been shown to influence severe COVID-19. Carriers of HLA-C*04:01 have two times the risk of needing automatic ventilation [82]. In addition, HLA-C*04:01 could be related to COVID-19 susceptibility. This was further assessed by genotyping of 12,139 Russian individuals from another cohort [75]. HLA-C*07:01 had a significant negative correlation with SARS-CoV-2 susceptibility and death rate [66,74], while HLA-C*01 and HLA-C*04:01 were positively associated with SARS-CoV-2 infectivity, severity, and death rate [47,58,100,101] Weiner [76] et al. noticed that HLA-C*04:01 is prevalent among British and Russians, but uncommon among the Taiwanese population [83]. In another study, HLA-C*07:01 was linked to a decreased risk of symptomatic COVID-19 [40]. The increased risk of hospitalization suggests that the HLA-C*04:01 allele increased the risk of COVID-19 severity. COVID-19-infected individuals with HLA-C*04:01, whose disease diagnosis was determined by days with the ventilator, were statistically significant to increased risk of COVID-19 after Bonferroni’s correction (*p* = 0.0023) [47]. In addition, another study with 92 COVID-19-infected individuals of 15 different nationalities with varying severity from the United Arab Emirates also observed a significant association between HLA-C*04 and COVID-19 severity (*p* = 0.0077) [65]. A study with 9373 COVID-19-infected individuals and 5943 controls in Spain showed that HLA-C*04:01 was linked with severe COVID-19 (*p* = 0.045) [48].

In a study, with 435 mild to severely symptomatic individuals from Spain (*n* = 133), Germany (*n* = 135), Switzerland (*n* = 20), and the US (*n* = 147), HLA-C*04:01 has been shown to have a potential association with severe COVID-19. SARS-CoV-2-infected HLA-C*04:01 carriers had two times more intubation risk (adjusted *p*-value = 0.0074). This could be due to other HLA alleles having more SARS-CoV-2 peptide binding sites than HLA-C*04:01. HLA-C*04:01 carriers are linked to SARS-CoV-2 severity, suggesting that HLA class I is involved in SARS-CoV-2 immune defense [76]. HLA-C*04:01 was higher in COVID-19-infected individuals than in healthy individuals [66]. The frequency of HLA-C*04:01 was about 13% in Germans, 15% in Spain, 19% in Germans with Turkish ancestry, and 16% in Switzerland [73]. The relationship between severe COVID-19 and HLA-C*04:01 remained when ethnicity was a covariate, and the effect of homogeneous populations was calculated [76]. Intubation was associated with HLA-C*04:01 (adjusted *p*-value = 0.0074) when applying age, gender, and ethnicity as covariates. An association was observed between intubation and HLA-C*04:01, with the exclusion of additional covariates (OR = 2.9), (adjusted *p*-value = 0.02) [76]. There was a chance that the correlation between COVID-19 severity and HLA-C*04:01 was a statistical artifact in one of their datasets. For individual ethnicities, an association between HLA-C*04:01 and severe COVID-19 was presented in every group. This association was insignificant, except for Caucasians, because of the small sample size. However, African Americans, Hispanics, and Caucasians who were HLA-C*04:01 carriers were admitted to the intensive care, and all Hispanics, African Americans, and 66% of Caucasians who were HLA-C*04:01 carriers underwent intubation [76]. The HLA-C*04:01 allele was recognized as a severe COVID-19 risk factor among 2113 individuals who disclosed that they were COVID-19-infected, and 10,026 individuals were controls from the cohort, Genotek. HLA-C*04:01 accounted for 13% of the allele frequency. HLA-C*04:01 enhanced the COVID-19 risk significantly in an association analysis with age, gender, and body mass index (BMI) as covariates (*p*-value = 0.005). The comprehensive effect of HLA-C*04:01 on severe COVID-19 was depicted by the odds ratio of 1.1 (*p*-value = 5.8 × 10^−4^) [76]. Weiner et al. (2021) showed that the HLA alleles could affect disease severity via unusual binding affinity between HLA and peptides of SARS-CoV-2. Following the Iturrieta-Zuazo approach, [102] showed the amount of SARS-CoV-2 peptides that “strongly” (at <50 mM) or “weakly” (at <500 mM) bound to the HLA allele. HLA-C*04:01 had one of the ten lowest HLA allele binding abilities to SARS-CoV-2 peptides. The late immune response triggered by low HLA binding affinity may be the reason for the severe COVID-19 in individuals with HLA-C*04:01 [76]. In addition, HLA-C*04:01 was linked with increased amounts of C-reactive protein (CRP) as an alternative for pervasive inflammation (Wilcoxon test, *p* = 0.021); however, the result was trivial (*r* = 0.2). Between ICU patients and non-ICU patients (*p* < 10^−5^) and intubation and non-intubation patients (*p* < 10^−5^), the CRP was significantly different [76]. HLA-C*04:01 could cause more dreadful consequences via more severe inflammation [103,104]. HLA-C*04:01 is a potential risk allele that was associated with double the intubation risk when one allele was present. These results were replicated in a COVID-19 shared dataset at Albany Medical Center, US [105], and data from the University of California, San Francisco, and the US. There was a strong association between HLA-C*04:01 and intubation. Furthermore, KIR2DS4 polymorphisms and HLA-C*04:01 increased the SARS-CoV-2 viral quantity and led to severe COVID-19 in individuals co-infected with HIV [106]. KIR2DS4f and HLA-C*04:01 combined were detected in four individuals, one of which was KIR2DS4-homozygous. This individual had severe COVID-19, had high troponin T hs, and was intubated [76]. There was no association between HLA-C*04:01 and the initial measure of viral load from patients during hospital admission [107]. It might be likely that patients’ viral load with HLA-C*04:01 might be increased throughout the initial stage of infection [108], but patients start to develop symptoms after 7 days [109,110,111]; this period may have been skipped to determine the relationship with SARS-CoV-2 viral loads and HLA-C*04:01 when recorded at the beginning of infection. Overall, no particular HLA allele correlated with the initially recorded viral load [76]. The HLA-C*04:01 allele had a significant association with COVID-19 susceptibility in the independent cohort. In these analyses, certain research could not find a correlation between HLA-C*04:01 and COVID-19 [76]. rs143334143 (CCHCR1) showed a significant association with COVID-19 severity. In the 1KG European cohort, HLA-C*04:01 was in linkage disequilibrium with the rs143334143 variant. Although, in another analysis [112], other SNPs in the same linkage disequilibrium category as HLA-C*04:01 and rs143334143 did not have the same effect. Conversely, there was significant heterogeneity between research papers in this analysis (*p*-value = 3.2 × 10^−3^) [76]. In an Armenian population of 299 COVID-19-infected individuals, HLA-C*04 was associated with a risk of hospitalization [113]. HLA-C*04:01 has been studied extensively in various populations and has shown some significant impact on COVID-19 disease and should be analyzed further as a potential therapeutic target.

## 4. Discussion

Differentiation in HLA expression levels has been formerly shown to be associated with infectious and autoimmune diseases, such as HIV, Parkinson’s, Crohn’s disease, and cancer [114], but there is a wealth of knowledge to be discovered about the relationship between HLA and COVID-19 [115,116,117], particularly HLA class I. This relationship should be analyzed in the future, as a similar trend could be detected with COVID-19.

SARS-CoV-2 variations can impact the course of infection, antigen presentation, and HLA binding [118]. An HLA allotype might present the most frequent peptide effectively, but a mutant strain differently. NetMHCpan tools are trained on binding affinity data [119,120]. Nersisyan et al. created tools to trace the SARS-CoV-2 mutation effect on HLA binding [119]. Delta, the highly contagious strain, and Omicron, the highly mutated, infective, and transmissible strain, are the most prevalent SARS-CoV-2 variants [121,122]. In spite of the results not directly correlating with T-cell responses, the data allow further research to hypothesize about these mutations. Genetic variations in the virus and HLA regions contribute to the effect of the presentation of antigens to T cells and ultimately affect the immune response and COVID-19 disease outcomes. While some polymorphisms might affect than others more, it is important to identify such genetic variations in therapeutics for current and future coronaviruses. While the viruses continue to mutate rapidly, it is more promising to put more focus on the host genetics to find effective and longer-lasting therapeutics. In addition, results might not correlate directly with the T-cell response because various other factors and genes contribute to disease outcomes. Related genes should be analyzed together with HLA to gather a more holistic view of COVID-19 pathogenesis.

Prugnolle et al. indicated that approximately 39% of HLA class I diversity is a consequence of human migration and is possibly pathogen-driven [123]. Thus, HLA diversity is known to increase with higher pathogen exposure [124]. As previously mentioned, Africa has one of the highest disease burden rates. Therefore, HLA diversity decreases outside of Africa. Hence, HLA types that are present in Africa are different than other countries or among other ethnicities [124]. TB in Western Europe and malaria in Africa have been shown to drive some of these pressures of diversity on immune-associated genes [125]. *HLA-C* diversity is unlikely to be due to viral pathogens. The most protective HLA class I is HLA-B*57. HLA-B*57:01 and HLA-B*57:03 are the most widespread subtypes in Caucasian and African populations, presenting protection against HIV disease progression [126,127]. This highlights the HLA allele differences and its effect on infectious diseases among different ethnicities. The genetic diversity in Africa is not fully understood. HLA-specific studies are imperative to fully understand the relationship between HLA and COVID-19 particularly in Africa.

The HLA molecule’s role is not fully understood, and more HLA-based studies are required. HLA and COVID-19 studies’ inconclusiveness and irreproducibility depend on the study groups, sample sizes, genetic variation, separation in phenotype definitions of selected alleles, HLA typing methods, and what type of studies are compared [36,45,46,64,72,102]. The different allele frequencies in studies vary between regions. Risk or protection alleles in certain findings have no relevance in other populations, because of their absence [89]. HLA and COVID-19 studies require bigger cohorts. Another limitation of these studies is that it is hard to determine the value of HLA in parallel with other disease risk considerations, like lifespan and co-existing conditions [128,129].

Evidence suggests that including HLA in clinical trials and joining COVID-19 testing with HLA typing to determine which factors are associated with disease severity in different populations. However, some consistent patterns between HLA and SARS-CoV-2 relationships can contribute to explaining antigen presentation in related studies. The forecasts from immunoinformatics show the binding affinity of the peptide of SARS-CoV-2 to *HLA-A* and in silico investigations have shown the relevance of HLA in the risk of SARS-CoV-2 and its importance in vaccine targets [42,47,61,83]. 

Despite Africa being highly burdened with infectious diseases, it has not been severely impacted by COVID-19 compared to other regions; researchers need to identify the host genetics in this region. In addition, the African population is the most genetically diverse among humankind [130]. Therefore, it is of utmost importance to focus on the impact of human genetics on infectious diseases in Africa. 

## 5. Conclusions

GWAS results from large populations reported COVID-19 severity associations [131,132,133], but more studies in an African population are required. These findings may provide new insights into the SARS-CoV-2 pathogenesis, identify high-risk individuals, and decrease mortality and morbidity. Identifying individuals at high risk of SARS-CoV-2 could assist with averting viral spread and reduce the health burden.

Identifying alleles involved in protection will increase the discovery of SARS-CoV-2 target epitopes, which will support forthcoming vaccine research [134]. Regrettably, the data from advanced countries may not be relevant to other regions. Research needs to focus on HLA allele frequency and SARS-CoV-2 mutation data globally to unravel this pandemic efficiently and prevent future pandemics.

## Figures and Tables

**Table 1:** HLA class I genes associated with COVID-19.

Gene	Ethnicity	Effect/Association	No. of Samples	*p* Value	Reference
*HLA-A*					
HLA-A*01	Mexican	Risk of fatal COVID-19	146 111 COVID-19-infected individuals and 35 controls	Pc = 0.03	[35]
	Spain	Associated with higher mortality	3886	Apache *p* = 0.04 or sofa *p* = 0.02	[36]
	West Indian	Prevalent in controls than COVID-19 patients	228 controls, 235 COVID-19 patients	*p* = 0.011	[37]
HLA-A*01:01	Russian	Protective against severe COVID-19	100 pneumonia caused by COVID-19 patients and 100 controls	*p* = 0.009	[38]
HLA-A*02	UK (Manchester and Leeds)	Might cause a protective effect or effective immune response against COVID-19 Protective against susceptibility and mortality	80 COVID-19-infected individuals 308 wait-listed renal transplants 10,000 deceased	*p* = 0.0179 Insignificant after correction	[39]
	West Indian	Prevalent among COVID-19-infected individuals of varying severity	228 controls, 235 COVID-19 patients	*p* < 0.001	[37]
HLA-A*02:01	German	Associated with symptomatic COVID-19	6919 COVID-19-infected individuals	*p* = 0.03	[40]
	Japanese	Low COVID-19 risk.	1336		[41]
	19 countries	Increased risk of COVID susceptibility and mortality.		*p* = 0.20	[42]
HLA-A*02:05	German	Associated with the risk of severe respiratory infection	6919 COVID-19-infected individuals	*p* = 0.04	[40]
HLA-A*26	Manchester and Leeds	Increased in patients than in controls	80 COVID-19-infected individuals, 308 wait-listed renal transplants, 10,000 deceased donors	*p* = 0.0049	[39]
HLA-A*02:06	Asia, North America, Europe, Oceania		6421 sequences		[41]
HLA-A*03	Prevalent in COVID-19 patients.		*p* = 0.047		
	Iranian	Associated with risk	142 COVID-19-infected individuals and 143 controls	*p* = 0.0025	[43]
HLA-A*03:01		Low COVID-19 risk			[44]
HLA-A*11	Spain	Higher mortality Increased frequency in deceased than in survivors	3886	SOFA (*p* = 0.04) APACHE (*p* = 0.02) *p* = 0.051	[36]
	Chinese	Possibly confers susceptibility in SARS-CoV-2 infection.	332 patients	* p * = 8.5 × 10^−3^	[45]
HLA-A*11:01	Japanese	Severe disease, hospitalization, and mortality COVID-19 severity Protective against COVID-19 susceptibility and mortality	190	*p* = 3.34 × 10^−3^ *p* = 0.013 *p* = 0.0078	[46]
	Albany, NY, USA	Increased risk of hospitalization	100 hospitalized COVID-19 patients and 26 controls	*p* = 0.0078	[47]
	Spanish	COVID-19 severity	5943 controls 9373 COVID-19-infected individuals	*p* = 0.033	[48]
HLA-A*23:01	Brazilian	Protection against COVID-19			[49]
HLA-A*24	Iranian		48 severe COVID-19 cases	*p* = 0.003	
HLA-A*24:02	Brazilian	Protection against COVID-19			[49]
	Ecuadorians	Protection against severe COVID-19			[50]
HLA-A*24:02:01	Chinese	Susceptibility	5		[51]
HLA-A*24:02	Japanese	Worse COVID-19 outcomes	1336		[41]
HLA-A*26:01	Russian	Worse COVID-19 outcome	111 COVID-19-infected individuals and 428 controls	*p* = 0.0459	[44]
	South Han Chinese	Associated with diabetes a risk factor for COVID-19	5		[51]
	Russian		111	0.0400	
HLA-A*30:02	African American	Increased COVID-19 susceptibility	234 COVID-19 cases and 22,000 controls	* p * = 0.01	[52]
	Albany, NY, USA	Enriched in COVID-19-positive individuals	100 hospitalized COVID-19-infected individuals and 26 controls	(Exact test) *p* = 0.0417	[47]
	Brazilian	Protection against COVID-19			[49]
HLA-A*31:01	Brazilian	Protection against COVID-19			[49]
HLA-A*32	Spain	Higher in healthy controls than COVID-19 patients	3886	(*p* = 0.004)	[36]
	Iranian	Protection against COVID-19	143 controls and 142 COVID-19-infected individuals	*p* = 0.0388	[43]
HLA-A*68	Iranian	Prevalent in COVID-19	48 severe COVID-19 and 500 controls	*p* = 0.001	[53]
	Mexican	Protective against severe COVID-19	146 111 COVID-19-infected individuals and 35 controls	PC = 0.03	[35]
HLA-A*68:01	Brazilian	Protection against COVID-19			[49]
*HLA-B*					
HLA-B*07	74 countries	Risk of mortality		*p* = 0.00081 Insignificant after multivariable regression	[54]
HLA-B*07:03	Hong Kong	Disease	90	* p * = 0.00072	[55]
HLA-B*08	Italians	Increased COVID-19 and death rate		HLA−A*01:01g−B*08:01g−C*07:01g−DRB1*03:01gG (*p* = 0.00042, *p* = 0.013)	[56]
HLA-B*08:01	74 countries	Increased COVID-19 and death rate	104,135	*p* = 0.047 (insignificant after multivariate regression with backward elimination)	[54]
HLA-B*08:01	209 populations		420 HLA-B alleles	<0.0001	[57]
HLA-B*14	Italians	Inversely associated with COVID-19	370,000	* p * < 0.0001	[58]
HLA-B*14:02	Chinese	Patients entering the severe stage.	332	* p * = 3 × 10^−3^	[59]
HLA-B*15	West Indian	Protection against COVID-19	228 controls, 235 COVID-19 patients	*p* = 0.008	[37]
	Egyptian	Protection	69	*p* < 0.001	[60]
HLA-B*15:03		Protective against COVID-19			[61]
HLA-B*15:01	805 district populations from 101 countries	Positively associated with COVID-19			[62]
		Asymptomatic SARS-CoV-2			[63]
*HLA-B1527*	Chinese	More frequent in COVID19-infected individuals than in healthy controls	82	*p* = 0.001	[59]
HLA-B*18	Italians	Inversely associated with COVID-19	370,000 and additional 120,926 individuals	* p * < 0.0001	[58]
HLA-B*18:01	Italian	Protects against COVID-19 incidence and mortality		HLA-A*02.01g-B*18.01g-C*07.01g-DRB1*11.04g (*p* = 0.0053, *p* = 0.034)	[56]
	Brazilian	Protection against COVID-19			[49]
HLA-B*22	Chinese	SARS-CoV-2 susceptibility	190 COVID-19-infected individuals and 294 controls	*p* = 0.032	[64]
HLA-B*27	Chinese	More prevalent among controls than COVID-19 patients Susceptibility and resistance to all SARS-CoV-2 strains	190 COVID-19-infected individuals and 294 controls	*p* = 0.068	[64]
HLA-B*35	United Arab Emirates (15 nationalities)	Severe COVID-19	92 patients	*p* = 0.0051	[65]
	South Asian	Severe COVID-19			
HLA-B*37:01	Russia	Associated with deceased COVID-19 individuals	111 COVID-19-infected individuals and 428 controls	*p* = 0.0331	[44]
HLA-B*38	Iranian	Disease susceptibility	48 severe cases of COVID-19	* p * < 0.001	[53]
HLA-B*39	Spain	Higher COVID-19 rates	72 infected out of 3886	*p* = 0.02	[62]
	Ecuadorian	Associated with COVID-19 risk	52 COVID-19-infected individuals and 87 controls		[50]
HLA-B*41	Egyptian	Associated with severe COVID-19	69		[60]
HLA-B*44	Italian	High risk for COVID-19 susceptibility, severity, and mortality in population-based studies.	182 patients, 619 controls	*p* = 0.175	[66]
	74 countries	Risk of mortality		*p* = 0.0022 (insignificant after multivariable regression)	[54]
	UK (Manchester and Leeds)	Protective effect	10,000 deceased donors, 308 wait-listed renal patients, and 80 COVID-19-infected individuals	*p* = 0.0052 (did not remain significant after correction).	[39]
HLA-B*44:02	60 countries 209 populations	High risk for COVID-19 susceptibility, severity, and mortality in population-based studies.	420 Hla-b alleles	0.0003	[57]
	Brazilian	Protection against COVID-19			[49]
	German	Associated with the risk of hospitalization	6919 COVID-19-infected individuals	*p* = 0.008	
HLA-B*46:01	Chinese, Vietnamese, Taiwan, Singaporean	Increased COVID-19 susceptibility			[67]
		Prevalent in mild COVID-19			[68]
HLA-B*49	Italians	Inversely associated with COVID-19	370,000 and additional 120,926 individuals	* p * < 0.0001	[58]
HLA-B*51	South Asian	Fatal COVID-19			[69]
HLA-B*52:01	African, European, Asian, Australian, Oceanian, American.		158 and 374 typed samples		[70]
HLA-B*54:01	Six areas (Asia, North America, South America, Europe, Oceania, and Africa)	SARS-CoV-2 susceptibility	158 and 374 typed samples		[70]
		Protection against COVID-19 infection	12,343 SARS-CoV-2	*p* = 0.017 (insignificant after adjusted *p* = 0.45)	[71]
HLA-B*55	Iranian	Protection	142 COVID-19 patients and 143 controls	*p* = 0.0033	[43]
HLA-B*55:01	African, European, Asian, Australian, Oceanian, American.	SARS-CoV-2 susceptibility	158 and 374 typed samples		[70]
HLA-B*55:07	African, European, Asian, Australian, Oceanian, American.	SARS-CoV-2 susceptibility	158 and 374 typed samples		[70]
HLA-B*55:12	African, European, Asian, Australian, Oceanian, American.	SARS-CoV-2 susceptibility	158 and 374 typed samples		[70]
HLA-B*51:01	Chinese	Severe COVID-19	332 patients	*p* = 0.007	[45]
	Brazilian	Protection against COVID-19			[49]
HLA-B*56:01	African, European, Asian, Australian, Oceania, American.	SARS-CoV-2 susceptibility	158 and 374 typed samples		[70]
	Hong Kong Chinese	Associated with COVID-19	190 COVID-19 cases	*p* = 0.045	[64]
HLA-B*56:04	Hong Kong Chinese	Associated with COVID-19	190 COVID-19 cases	*p* = 0.029	[64]
HLA-B*58	Iranian	Protection	143 controls and 142 COVID-19-infected individuals	*p* = 0.0376	[43]
	74 countries	Risk of death		* p * = 0.0089 (insignificant after adjustment).	[54]
HLA-B*58:01	209 populations	High risk for COVID-19 susceptibility, severity, and mortality in population-based studies.		0.0062	[57]
	Italian	Positively associated with COVID-19	99 patients	* p * = 0.01317	[72]
*HLA-C*					
HLA-C*01		Prevalent in mild COVID-19 infection compared to severe COVID-19	228 controls, 235 COVID-19 patients	*p* = 0.004	[37]
	Italian	Permissive to SARS-CoV-2. More prevalent in the deceased than in survivors.		*p* = 0.09	[36,58]
HLA-C*03	Italian	Positively associated with the incidence of SARS-CoV-2 infection	370,000 individuals and an additional 120,926 individuals	*p* > 0.0001	[58]
	Saudi	Increased COVID-19 severity	136 COVID-19 patients	*p* = 0.047	[69]
HLA-C*04:01	Germany, Spain, Switzerland, and the United States	Severe COVID-19	435	*p* = 0.0074	[73]
	Europeans	Severe COVID-19	619 controls and 182 infected individuals.		[74]
	Russian	Associated with COVID-19 susceptibility.	12,139		[75]
	Sardinian	Susceptibility to SARS-CoV-2 infection	619 controls, 182 SARS-CoV-2 patients	*p* = 0.001	[66]
		Increased risk of COVID-19		*p* = 0.005	[76]
	Albany, NY, USA	Severe COVID-19	100 hospitalized COVID-19 infections and 26 controls	*p* = 0.0087	[47]
		Increased risk of hospitalization measured by days with ventilation		*p* = 0.0023	
	United Arab Emirates (15 nationalities)	COVID-19 severity	92 COVID-19-infected individuals	*p* = 0.0077	[65]
HLA-C*05	Spain	Severe COVID-19	9373 COVID-19-infected individuals and 5943 controls	*p* = 0.045	[48]
	74 countries	Risk of COVID-19 death		*p* = 0.000027	[54]
HLA-C*05:01	Brazilian	COVID-19 protection			[49]
HLA-C*06:02	Manchester and Leeds	Worse COVID-19 disease outcome	80 infected out of 308		[39]
HLA-C*7	Egyptian	Associated with protection from death	69 COVID-19 patients	*p* = 0.001	[60]
HLA-C*07:01	Sardinian	Negatively correlates with SARS-CoV-2 susceptibility and mortality	619 controls, 182 SARS-CoV-2 patients	*p* = 0.0406	[66]
	Brazilian	COVID-19 protection			[49,77]
	German	Decreased risk of symptomatic COVID-19	6919 COVID-19-infected individuals	*p* = 0.001	[40]
HLA-C*07:29	Chinese	Higher expression in COVID-19 patients than controls.	82	*p* = 0.001	[59]
HLA-C*08:02	Sardinian	Increased susceptibility SARS-CoV-1	619 controls, 182 SARS-CoV-2 patients	HLA-A*30:02, HLA-B*14:02, and HLA-C*08:02 haplotypes (*p* = 0.0008)	[66]
	Brazilian	COVID-19 protection			[49]
	Spanish Mediterranean Caucasian	Mild COVID-19		*p* = 0.0014	[48]
	Spanish	Reduced risk of COVID-19	9373 COVID-19 positive cases and 5943 controls	*p* = 0.024	[48]
HLA-C*12	Manchester and Leeds	Prevalent in the control population	80 COVID-19-infected, 308 wait-listed renal transplants (control), and 10,000 deceased donors (control)	*p* = 0.0286	[39]
	Egyptian	Protection from death	69 COVID-19 patients	*p* = 0.008	[60]
HLA-C*12:02	Han	High response against COVID-19	5		[51]
HLA-C*12:03	Spanish Mediterranean Caucasian	Mild COVID-19 compared to critical COVID-19	72 individuals, 24 COVID-19-infected individuals, and 48 hospitalized	*p* = 0.0001	[78]
HLA-C*14:02	Chinese	Severe COVID-19	332	*p* = 0.003	[45]
HLA-C*15:02	Brazilian	COVID-19 protection			[49]
HLA-C*16	Egyptian	COVID-19 severity	69 COVID-19 patients		[60]
	Spain	Increased COVID-19 infection	3886	*p* = 0.02	[36]
HLA-C*16:01	Spanish Mediterranean Caucasian population	Associated more with mild COVID-19 when compared to critical than severe	72 individuals, 24 COVID-19-infected individuals, and 48 hospitalized	*p* = 0.0014	[78]
HLA-C*17	Egyptian	COVID-19 severity	69 COVID-19 patients		[60]
HLA-C*17:01	Brazilian	Associated with COVID-19 protection			[49,77]

* The asterisk allows for differentiation between variants within the same HLA gene group.

## Data Availability

Not applicable.
